# Persistent organic pollutant exposure contributes to Black/White differences in leukocyte telomere length in the National Health and Nutrition Examination Survey

**DOI:** 10.1038/s41598-022-24316-0

**Published:** 2022-11-19

**Authors:** Emily K. Roberts, Jonathan Boss, Bhramar Mukherjee, Stephen Salerno, Ami Zota, Belinda L. Needham

**Affiliations:** 1 Department of Biostatistics, University of Michigan, Ann Arbor, MI 48109-2029 USA; 2 Department of Epidemiology and Center for Social Epidemiology and Population Health, University of Michigan, 1415 Washington Heights, 4659 SPH Tower, Ann Arbor, MI 48109-2029 USA; 3 Department of Environmental and Occupational Health, George Washington University Milken School of Public Health, Washington, USA; 4 Department of Environmental Health Sciences, Columbia University Mailman School of Public Health, New York, USA

**Keywords:** Environmental sciences, Biomarkers

## Abstract

Despite racial disparities in diseases of aging and premature mortality, non-Hispanic Black Americans tend to have longer leukocyte telomere length (LTL), a biomarker of cellular aging, than non-Hispanic White Americans. Previous findings suggest that exposure to certain persistent organic pollutants (POPs) is both racially-patterned and associated with longer LTL. We examine whether Black/White differences in LTL are explained by differences in exposure to 15 POPs by estimating the indirect effect (IE) of self-reported race on LTL that is mediated through nine polychlorinated biphenyls (PCBs), three furans, and three dioxins, as well as their mixtures. Our study population includes 1,251 adults from the 1999–2000 and 2001–2002 cycles of the cross-sectional National Health and Nutrition Examination Survey. We characterized single-pollutant mediation effects by constructing survey-weighted linear regression models. We also implemented various approaches to quantify a global mediation effect of all POPs, including unpenalized linear regression, ridge regression, and examination of three summary exposure scores. We found support for the hypothesis that exposure to PCBs partially mediates Black/White differences in LTL. In single-pollutant models, there were significant IEs of race on LTL through six individual PCBs (118, 138, 153, 170, 180, and 187). Ridge regression (0.013, CI 0.001, 0.023; 26.0% mediated) and models examining summative exposure scores with linear combinations derived from principal components analysis (0.019, CI 0.009, 0.029; 34.8% mediated) and Toxic Equivalency Quotient (TEQ) scores (0.016, CI 0.005, 0.026; 28.8% mediated) showed significant IEs when incorporating survey weights. Exposures to individual POPs and their mixtures, which may arise from residential and occupational segregation, may help explain why Black Americans have longer LTL than their White counterparts, providing an environmental explanation for counterintuitive race differences in cellular aging.

## Introduction

Non-Hispanic Black Americans have longer leukocyte telomere length (LTL) than non-Hispanic White Americans, despite having greater exposure to risk factors for short LTL (e.g., low socioeconomic status (SES) and interpersonal discrimination), as well as increased risk of health-related outcomes associated with short telomeres (e.g., cardiovascular disease and premature mortality)^1^. While some have argued that race differences in LTL are due, at least in part, to genetic variation^2–4^, emerging evidence reviewed below (and discussed in^1^) points to a potential environmental explanation for the counterintuitive finding that Black Americans have longer LTL than White Americans. Using data from the National Health and Nutrition Examination Survey (NHANES), we examine whether Black/White differences in LTL are partially mediated by differences in exposure to persistent organic pollutants.

### Persistent organic pollutants and LTL

Telomeres are the protective caps at the ends of chromosomes that promote chromosomal stability^5^. Due to the end replication problem, telomeres shorten every time a cell divides^6^. DNA replication stress and oxidative damage also contribute to telomere shortening^5,7^. Telomerase can counteract shortening by elongating and protecting telomeres^8^, but this enzyme is kept downregulated in normal human cells^5^. Once telomeres become critically shortened, cellular senescence is triggered, causing cells to lose the ability to grow and divide^9,10^. Thus, telomere shortening is considered a hallmark indicator of cellular aging^11,12^. Recent Mendelian randomization studies suggest that short LTL may be a causal determinant of degenerative diseases, including cardiovascular disease and Alzheimer’s disease, while long LTL may be a causal determinant of some types of cancer, including lung, bladder, endometrial, and testicular cancer^13–21^.

A growing body of epidemiologic and experimental evidence suggests that LTL can be a sensitive endpoint for environmental chemicals, such as persistent organic pollutants (POPs), that are capable of promoting carcinogenesis. Polychlorinated biphenyls (PCBs), furans, and dioxins are ubiquitous POPs that bioconcentrate in the food chain and accumulate in adipose tissue. PCBs were once widely manufactured and used as coolants or lubricants, whereas furans and dioxins are unintentionally produced as industrial byproducts in a variety of commercial and industrial settings. Although PCB production and use were banned in the mid-1970s in the United States, most people continue to be exposed at low levels due to the long half-lives of these chemicals both in the environment and in the human body. Human exposure to PCBs can occur by living close to PCB-contaminated waste sites, ingestion of contaminated food, ingestion and/or inhalation of contaminated indoor dust and air, and from occupational environments^22^. Furans and dioxins are currently regulated as hazardous air pollutants, but they continue to be produced during most forms of combustion, including burning of municipal and medical waste and as part of industrial processes^23^.

To date, most epidemiologic studies have observed a positive association between exposure to POPs and LTL. For example, previous research using nationally representative data from NHANES has shown that exposure to PCBs, which are classified as human carcinogens by the International Agency for Research on Cancer^22,24^, is associated with longer LTL^25–28^. These cross-sectional associations were observed both for individual chemicals (i.e., PCB congeners, dioxins, furans) as well as their mixtures^27^. Associations between POPs and longer LTL have also been observed in more highly exposed populations, such as residents of Anniston, Alabama, where PCBs were historically manufactured^29,30^. Population-based studies in Korea^31^ and Iran^32,33^ have reported similar findings. In contrast to these results, some previous studies have found that exposure to POPs is associated with shorter LTL. Age-adjusted median LTL in lymphocytes, but not granulocytes, was shorter in German workers occupationally exposed to PCBs compared to healthy controls^34^. In the only longitudinal study to date, serum concentrations of PCB 153 were associated with increased relative LTL shortening over ten years of follow-up in a subset of elderly respondents from the Helsinki Birth Cohort^35^.

A recent experimental study is consistent with the epidemiologic evidence suggesting that exposures to PCBs and dioxins are associated with longer LTL. In the only in vivo study to date, researchers found that exposure to 2,3,7,8-tetrachlorodibenzo-p-dioxin (TCDD) and several PCB congeners, both alone and in combination, resulted in an increased LTL. Specifically, rats exposed to PCBs and TCDD had increased LTL in their liver and lung tissues as well as altered expression of genes related to telomere maintenance in their livers^36^.

Although the mechanisms linking exposure to POPs with longer LTL have yet to be fully elucidated, previous in vitro studies suggest that upregulation of telomerase may play a role. Telomerase is an enzyme that elongates telomeres and is hypothesized to confer uncontrolled replicative ability on a cell^37^. Two previous studies found that exposure to PCBs activates the proto-oncogene c-myc^38,39^, which is involved in activation of the telomerase reverse transcriptase (TERT) gene^40^. In another study, expression of TERT was upregulated in human choriocarcinoma cells treated with dioxin^41^. Drawing on previous work demonstrating that dioxins are potent aryl hydrocarbon receptor (AhR) agonists^42^, the authors suggested that AhR activation may mediate the association between exposure to dioxins and upregulation of telomerase^41^.

### Race differences in exposure to carcinogens

Evidence from multiple sources indicates that Black Americans have greater exposure to POPs than White Americans. Analysis of nationally representative data from NHANES indicates that Black participants have the highest PCB exposures of all racial/ethnic groups, and racial disparities in exposure are particularly pronounced for older adults who may have been exposed before the ban on production and use^43^. While fish consumption is an important source of PCB exposure in the general population, this exposure route does not explain the relatively high PCB body burden in Black adults^44^. Among 765 adults from Anniston, Alabama, where PCBs were historically manufactured, the geometric mean of the summed PCB congeners was more than 2.5 times higher for Black participants than for White participants (866 ng/g lipid vs. 331 ng/g lipid); this difference was not explained by age, sex, body mass index (BMI), or smoking status^30^. Results from the Anniston Community Health Survey also revealed that Black participants had a significantly higher average total dioxin toxic equivalencies (TEQ) score than White participants (33.1 vs. 19.2 pg/g lipid) after adjusting for age and sex^45^. Finally, in a preliminary analysis of data from the NIH-AARP Diet and Health Study linked with the US EPA database of 4,478 historical sources of polychlorinated dibenzo-p-dioxins and dibenzofurans (PCDD/F), investigators found that Black Americans were nearly three times as likely as White Americans to live within 5 km of a PCDD/F emitting facility^46^.

These data suggest that social-structural drivers may contribute to racial disparities in POP exposures. Residential segregation in the US remains deeply entrenched, despite laws prohibiting discrimination in housing on the basis of race^47^, and residents of predominantly Black neighborhoods are significantly more likely to be exposed to environmental hazards, including carcinogens^48,49^. Research suggests that Black Americans with high SES are only slightly less likely than those with low SES to live in residentially segregated, low-income areas^50,51^. Previous research has also shown that Black Americans are more likely to be exposed to carcinogens at work^52,53^.

### Hypotheses

This study brings together three separate lines of evidence to examine the hypothesis that Black/White differences in LTL are explained by differences in exposure to carcinogens. First, previous research has consistently shown that Black Americans have longer LTL than White Americans, although the reasons why remain unclear (see Needham et al.^1^ for a review). Next, there is growing evidence that exposure to POPs is associated with longer LTL^26–33^. Finally, prior studies have found that Black adults have greater exposure to POPs than White adults^35,44–46^. We use mediation analysis to quantify the indirect effect of race/ethnicity on LTL through exposure to PCBs, furans, and dioxins. We present single mediator models, followed by five multivariate-mediator methods, including four that use different approaches to account for the highly correlated nature of environmental chemicals.

## Methods

### Data collection and study population

NHANES consists of cross-sectional national surveys conducted by the U.S. Centers for Disease Control and Prevention (CDC) to monitor the health and nutritional status of the population^55^. NHANES provides information about their Ethics Review Board Approval, and all experimental protocols were approved by the Institutional Review Board at the CDC^55^. Informed consent was obtained during the interviews, and all methods were carried out in accordance with relevant guidelines and regulations**.** Detailed documentation for the NHANES protocol and methods can be found online. We use data from the 1999–2000 and 2001–2002 cycles of NHANES for this secondary data analysis. Data are anonymized by NHANES prior to use, and the data are freely available for the public. Because our study used a publicly available data set that does not include information that can be used to identify individuals, it is not considered human subjects research and, therefore, does not require approval from the University of Michigan IRB.

NHANES uses a complex, multistage, probability sampling design to obtain a sample that is representative of the US civilian noninstitutionalized population. Older adults (aged 60+) and Black and Hispanic Americans are oversampled in order to produce reliable statistics for these groups. NHANES 1999–2002 includes 21,004 respondents aged two months and older. PCBs, furans, and dioxins were measured in a subset of 4821 respondents. For this analysis, we excluded respondents whose self-reported race/ethnicity was not Black or White (n = 1743) or who were under the age of 20 (n = 910), since DNA samples are only available for those aged 20 and older. We also removed participants with at least one missing exposure among the subset of POPs of interest (n = 838; described further below). Respondents with missing blood cell count and distribution variables (n = 8), serum cotinine (n = 13), LTL (n = 57), and education (n = 1) were also removed. Our final analytic sample consists of 1,251 (321 Black and 930 White) study participants (see Supplemental Fig. 1).

### Measures

#### Outcome variable: telomere length (*Y*)

Aliquots of purified DNA from respondents who consented specifically to future genetic research were provided by the laboratory at the Division of Health and Nutrition Examination Surveys, National Center for Health Statistics, Centers for Disease Control and Prevention. Five 96-well quality control plates representing 5% of the complete set were also provided, and duplicate samples were blinded. DNA was extracted from purified whole blood using the Puregene (D-50 K) kit protocol (Gentra Systems, Inc., Minneapolis, Minnesota) and stored at –80° C. The telomere length assay was performed in the Blackburn Laboratory at the University of California, San Francisco, using the quantitative polymerase chain reaction (qPCR) method to measure telomere length relative to standard reference DNA (T/S ratio)^56,57^.

The telomere thermal cycling profile consisted of cycling for T (telomic) PCR: 96 °C for 1 min; denature at 96 °C for 1 s, anneal/extend at 54 °C for 60 s, with fluorescence data collection, 30 cycles. Cycling for S (single copy gene) PCR consisted of the following: 96 °C for 1 min; denature at 95 °C for 15 s, anneal at 58 °C for 1 s, extend at 72 °C for 20 s, 8 cycles; followed by denature at 96 °C for 1 s, anneal at 58 °C for 1 s, extend at 72 °C for 20 s, hold at 83 °C for 5 s with data collection, 35 cycles. The primers for the telomere PCR were tel1b [5′-CGGTTT(GTTTGG)_5_GTT-3′], used at a final concentration of 100 nM, and tel2b [5′-GGCTTG(CCTTAC)_5_CCT-3′], used at a final concentration of 900 nM. The primers for the single-copy gene (human beta-globin) PCR were hbg1 (5′ GCTTCTGACACAACTGTGTTCACTAGC-3′), used at a final concentration of 300 nM, and hbg2 (5′-CACCAACTTCATCCACGTTCACC-3′), used at a final concentration of 700 nM. The final reaction mix contained 20 mM Tris-hydrochloride (HCl), pH 8.4; 50 mM potassium chloride (KCl); 200 μM each deoxynucleotide (dNTP); 1% dimethyl sulfoxide (DMSO); 0.4 × SYBR Green I; 22 ng Escherichia coli DNA per reaction; and 0.4 units of Platinum Taq DNA polymerase (Invitrogen Inc.) per 11-μL reaction.

Each sample was assayed three times on three different days. Samples were assayed on duplicate wells, producing six data points. Sample plates were assayed in groups of three plates, and no two plates were grouped together more than once. Each assay plate contained 96 control wells with eight control DNA samples. Assay runs with eight or more invalid control wells were excluded from further analysis (< 1% of runs). Control DNA values were used to normalize between-run variability. Runs with more than four control DNA values falling outside 2.5 standard deviations from the mean for all assay runs were excluded from further analysis (< 6% of runs). For each sample, potential outliers were identified and excluded from further analysis (< 2% of samples). Finally, the mean and standard deviation of the T/S ratio were calculated normally, excluding potential outliers. DNA samples were coded, and the lab was blinded to all other measurements in the study. The CDC conducted a quality control review before linking the telomere data to the NHANES public use data files. If more than 5% of the duplicate samples on the quality control plates were discordant with their pair in the complete set, then the variant failed quality control^58^. The measure of LTL used in this study is an average across all leukocyte cell types, and LTL is log-transformed prior to analysis.

#### Exposure variable: race/ethnicity (*A*)

Race and ethnicity were self-reported and NHANES staff recoded these into five categories: non-Hispanic Black, non-Hispanic White, Mexican American, other Hispanic, and other race, including multi-racial. In line with previous work^59,60^, we consider self-reported race/ethnicity to be a variable that reflects an individual’s position within the racialized social hierarchy. As such, it is an important determinant of exposure to a broad array of health-related risks and protective factors, including exposures related to environmental racism. Though racial and ethnic categories are socially constructed, we acknowledge that these categories may also reflect differences in genetic ancestry. We chose to limit this analysis to individuals who self-identify as Black or White because differences in LTL by race/ethnicity are greatest for these groups and because the counterintuitive Black/White differences have yet to be explained.

#### Mediator variables: PCBs, furans, and dioxins (*M*)

Analytic methods for quantification of PCBs, furans, and dioxins in serum have been described in detail elsewhere^26^. Briefly, congeners were extracted from serum specimens using a C18 solid phase extraction and measured using high-resolution mass gas chromatography/spectrometry^61,62^. The analytical runs for each chemical were blinded and included quality control samples. Limits of detection (LOD) were typically around 2 ng/g but varied by serum volume. The LOD range for each chemical is reported in Mitro et al.^26^ The NHANES dataset includes flag variables indicating whether each observation was above or below the sample specific limit of detection. Since NHANES does not provide a single lipid concentration variable for each individual, we calculated serum lipids using Phillip’s short formula based on cholesterol and triglycerides data^63^.

#### Adjustment covariates (*Z)*

We considered adjustment for the following potential confounders of the exposure-outcome (*A-Y)* and mediator-outcome (*M-Y)* relationships: standardized age (linear and quadratic terms), sex, educational attainment, serum cotinine (log-scale), and lipids (log-scale)^64^. We also adjusted for white blood cell count, percent lymphocytes, percent neutrophils, percent eosinophils, percent basophils, and percent monocytes. Finally, due to potential batch effects across survey years, we controlled for the survey cycle (1999–2000 vs. 2001–2002) in all models.

### Statistical analysis

#### Multiple imputation procedure

The NHANES dataset includes an indicator variable for whether the concentration was above or below the LOD. In this analysis, we excluded environmental chemicals when more than 50% of the observations were below their respective detection limits in either the 1999–2000 or the 2001–2002 cycle. Thus, we considered 17 chemicals with less than 50% below their respective detection limits in the following analyses (as was done for PCBs in Mitro et al.^26^). Given the high percent of non-detects, we performed multiple imputation instead of using the simple imputation of non-detects with LOD divided by square root of two, which was provided by the CDC. Non-detects for the 17 environmental chemicals were multiply imputed using an iterative application of censored likelihood multiple imputation^65^. The imputation strategy generated 10 imputed datasets for each survey cycle separately to account for potential batch effects between the two NHANES cycles. Environmental chemicals were ordered by the percent below their respective detection limits in the 1999–2000 cycle and imputed sequentially from the lowest percent below LOD to the highest percent below LOD. In the imputation and mediation models described next, all chemicals were log-transformed^66^. Imputation models for each log-transformed environmental chemical were conditional on log-transformed LTL, race, education, age, sex, log-transformed serum cotinine, log-transformed blood composition variables and lipids, and all previously imputed, log-transformed environmental chemicals. Imputation quality for each chemical was visually assessed. Of the 17 imputed environmental chemicals, the final two imputed chemicals, PCB 156 and PCB 99, had unusually low imputed concentrations in the 1999–2000 cycle. We therefore decided to exclude PCB 156 and PCB 99 from all analyses. This left 15 total environmental chemicals for all subsequent analyses.

#### Descriptive statistics and exploratory analysis

We started by summarizing the marginal distributions of LTL, environmental chemicals, and covariates, in the full sample and stratified by race. The goal of exploratory analyses stratified by race was to check that the unadjusted associations between variables of interest and race obtained from our study population were concordant with associations previously reported in the literature. Next, we conducted exploratory analysis of the existing correlation structure among the POPs. The non-negligible pairwise correlations between the POPs are seen in the heat map shown in Supplemental Fig. 2, which suggest that an appropriate approach to mediation will need to handle multicollinearity issues.

#### Single mediator approach

**Figure 1:**
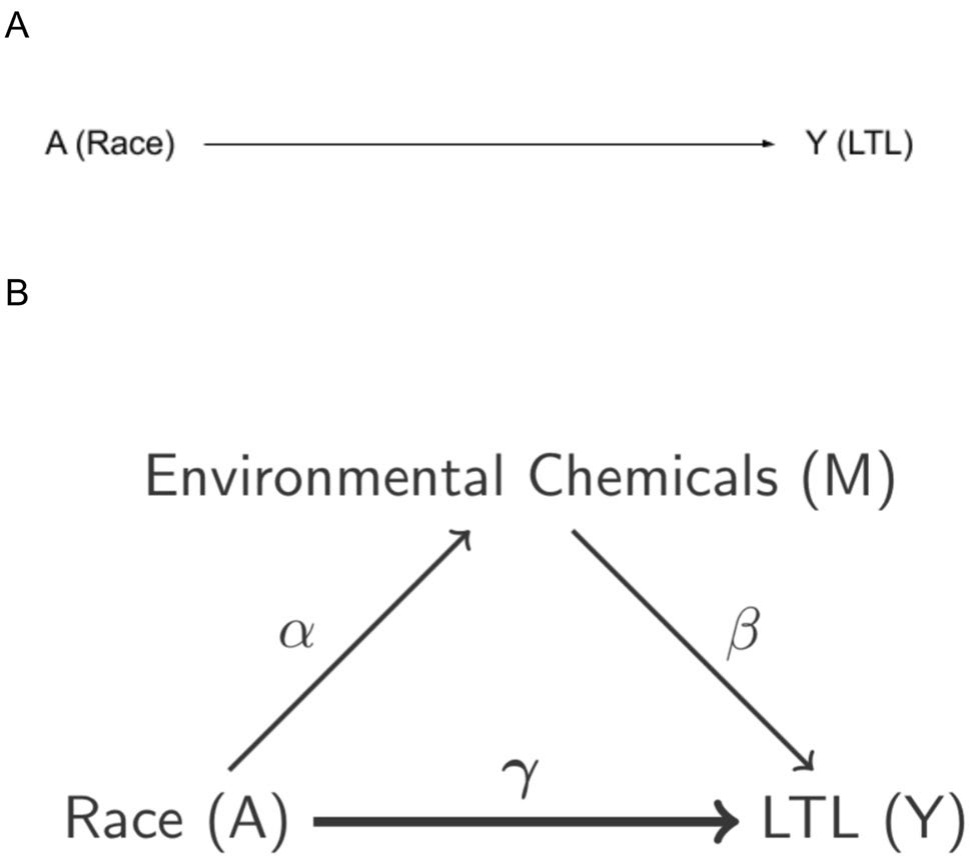
Conceptual models being considered in this work. Panel (**A**) shows the total effect model (conditional on adjustment covariates). In panel (**B**) we show the mediation model. The bolded line and corresponding coefficient show the direct effect. ${\alpha }_{aj}$ and ${\beta }_{mj}$(*j* = 1,…, 15 contaminants) show the relationships between race and the chemical and the chemical and LTL for each chemical respectively.

Our primary analytical goal was to estimate the indirect effect of race on LTL that is mediated through PCB, furan, and dioxin exposure (Fig. 1). First, we assessed the relationship between race and LTL to ensure there existed a non-null total effect (direct effect + indirect effect). More rigorously, we fit the *Y|A,Z* model, ${Y}_{i} = {\mu }_{0} + {\mu }_{a }{A}_{i} + {{\varvec{Z}}}_{{\varvec{i}}}^{{\varvec{T}}}{{\varvec{\mu}}}_{\boldsymbol{ }{\varvec{Z}}}+ {\epsilon }_{i\mu }$, where ${Y}_{i}$ is log-transformed LTL for the $i$-th individual, ${A}_{i}$ is the binary indicator of Black/White race for the $i$-th individual, ${{\varvec{Z}}}_{{\varvec{i}}}$ is the vector of adjustment covariates for the $i$-th individual, and ${\epsilon }_{i\mu }\sim N\left(0,{\sigma }_{\mu }^{2}\right)$ is the residual error for the $i$-th individual. We tested whether ${\mu }_{a}$, the total effect of race, is significantly different from zero using a Wald test. The direct and indirect effect estimates were then used to ascertain how much of the race and LTL association is explained through exposure to PCBs, furans, and dioxins.

To calculate the direct and indirect effects, we used the approach of Baron and Kenny^67^. We first constructed single mediator mediation models for race and each environmental mediator separately, adjusting for the potential confounding variables and adjustment covariates. More specifically, we first constructed the *M|A,Z* models, which are of the form ${M}_{ij }={\alpha }_{0j} + {\alpha }_{aj }{A}_{i}+{{\varvec{Z}}}_{{\varvec{i}}}^{{\varvec{T}}}{\boldsymbol{\alpha }}_{{\varvec{z}}{\varvec{j}}} + {\epsilon }_{ij\alpha }$ for the $j$-th environmental mediator, where ${M}_{ij}$ denotes the $j$-th environmental mediator for the $i$-th individual and ${\epsilon }_{ij\alpha }\sim N\left(0,{\sigma }_{j\alpha }^{2}\right)$ is the residual error for the $i$-th individual and $j$-th environmental mediator. The second set of models were the *Y|M,A,Z* models, which are of the form ${Y}_{i} = {\beta }_{0j} + {\beta }_{aj }{A}_{i}+{\beta }_{mj}{M}_{ij} + {{\varvec{Z}}}_{{\varvec{i}}}^{{\varvec{T}}}{{\varvec{\beta}}}_{{\varvec{z}}{\varvec{j}}}+ {\epsilon }_{ij\beta }$ for the $j$-th environmental mediator, where ${\epsilon }_{ij\beta }\sim N(0,{\sigma }_{j\beta }^{2})$ is the residual error for the $i$-th individual. Substituting the *M|A,Z* models into the corresponding *Y|M,A,Z* models, the total effect can be expressed as, ${\mu }_{a}={\beta }_{aj}+{\alpha }_{aj}{\beta }_{mj}$, where ${\beta }_{aj}$ denotes the direct effect of the $j$-th environmental mediator and ${\alpha }_{aj}{\beta }_{mj}$ denotes the indirect effect of the $j$-th environmental mediator. Sobel’s tests with a Benjamini–Hochberg correction to adjust for multiple testing were used to determine whether the indirect effect estimates ${\widehat{\alpha }}_{aj}{\widehat{\beta }}_{mj}$ were significantly different from zero, providing evidence that part of the total effect is mediated through environmental mediator $j$^68,69^. Moreover, we also calculated the percent mediated as $100\times {\alpha }_{aj}{\beta }_{mj}/{\mu }_{a}$. The *Y|A,Z* model, the *M|A,Z* models, and the *Y|M,A,Z* models all accounted for the NHANES sampling design, including survey weights and stratified cluster sampling, using the survey package in R^70^.

Note that, in order for the direct and indirect estimates from the Baron and Kenny method to have a causal interpretation, the following assumptions must hold^71,72^:

1. No unmeasured A $\to$ Y (race-telomere) confounding.
2. No unmeasured M $\to$ Y (POPs-telomere) confounding conditional on A.
3. No unmeasured A $\to$ M (race-POPs) confounding.
4. No M $\to$ Y confounder that is caused by A (POP-telomere caused by race).

We will discuss the plausibility of these assumptions and the interpretation of our results in the causal context in the discussion section. The assumption of no exposure-mediator interaction is not strictly required for estimation of indirect effects, though we explore this relationship in sensitivity analyses.

#### Multivariate mediation methods

Following the single mediator analysis, we then considered multiple multivariate mediation analysis methods, which quantify a global mediation effect of all POPs (see Table 2 for a comparison of all multivariate mediation methods used in this article). The most common of these approaches involves fitting an unpenalized linear regression outcome model using least squares, ${Y}_{i} = {\beta }_{0} + {\beta }_{a }{A}_{i}+{{\varvec{M}}}_{{\varvec{i}}}^{{\varvec{T}}}{{\varvec{\beta}}}_{{\varvec{m}}} + {{\varvec{Z}}}_{{\varvec{i}}}^{{\varvec{T}}}{{\varvec{\upbeta}}}_{\mathbf{z}}+{\epsilon }_{i\beta }$, along with the mediator model ${{\varvec{M}}}_{{\varvec{i}}\boldsymbol{ }}={\boldsymbol{\alpha }}_{0\boldsymbol{ }}+{A}_{i} {\boldsymbol{\alpha }}_{{\varvec{a}}\boldsymbol{ }}+{\boldsymbol{\alpha }}_{{\varvec{z}}}{{\varvec{Z}}}_{{\varvec{i}}} + {{\varvec{\epsilon}}}_{{\varvec{i}}\boldsymbol{\alpha }}$, where ${{\varvec{M}}}_{{\varvec{i}}}$ is a vector of candidate environmental mediators for the $i$-th individual, ${\epsilon }_{i\beta }\sim N(0,{\sigma }_{\beta }^{2})$, and ${{\varvec{\epsilon}}}_{{\varvec{i}}\boldsymbol{\alpha }\boldsymbol{ }}\sim MVN(0,{\boldsymbol{\Sigma }}_{{\varvec{m}}})$. Here ${\boldsymbol{\alpha }}_{{\varvec{a}}}^{{\varvec{T}}}{{\varvec{\beta}}}_{{\varvec{m}}}$ is the indirect effect $, {\beta }_{a}$ is the direct effect, and ${\beta }_{a }+{\boldsymbol{\alpha }}_{{\varvec{a}}}^{{\varvec{T}}}{{\varvec{\beta}}}_{{\varvec{m}}}$ is the total effect where ${\boldsymbol{\alpha }}_{0}$ and ${\boldsymbol{\alpha }}_{{\varvec{a}}}$ are vectors and ${\boldsymbol{\alpha }}_{{\varvec{z}}}$ is a matrix. However, this approach does not assuage variance inflation induced by the highly collinear structure of serum POP concentrations in the outcome model. To address the issue of variance inflation, we next considered a ridge-penalized linear regression outcome model, which utilizes a penalty term to shrink estimated coefficients toward zero^73^. For both of these outcome models, there were several challenges. One challenge specific to the ridge-penalized outcome model is the lack of consensus for obtaining analytical inferential quantities such as confidence intervals and p-values when the tuning parameter in the ridge penalty is selected via cross-validation. To circumvent this issue, we used the bootstrap to obtain confidence intervals and p-values for both the unpenalized outcome model and the ridge-penalized outcome model. The other major issue is that, to our knowledge, there is no existing R software to implement multivariate regression models fully adjusted for the complex survey design of NHANES. Therefore, we were not able to account for complex survey design for these two methods.

#### Environmental mediator summary scores

To deal with the challenge of incorporating survey design elements, we considered three additional analytical methods based on summary score constructions, where a linear combination of the environmental chemicals was used as the mediating variable. By collapsing the information contained in all environmental chemical measures into summary score metrics, the scores were then used as candidate mediators in the single mediator framework to fully incorporate the NHANES complex survey design. The purpose of constructing three distinct environmental chemical scores was to check the sensitivity of the results to different methods of constructing the summary scores. The environmental chemical scores were defined as a linear combination of the chemical concentrations ${\sum }_{j=1}^{15}{w}_{j}{M}_{ij}$, where the weights ${w}_{j}$ were determined using the following three approaches (i) principal components analysis (PCA), (ii) the first principal direction of mediation (PDM)^74^, and (iii) the Toxic Equivalency Quotient (TEQ) score.

PCA is a dimension reduction technique that constructs linear combinations of environmental chemicals such that the linear combinations are uncorrelated with one another. Each linear combination is called a principal component, with the first principal component explaining the most variability. All subsequent principal components explain the most remaining variability that is unexplained by the previous principal components. The weights for the PCA-based score were determined by the first principal component in the environmental chemical space. PDM is similar to PCA, with the difference being that the first principal direction of mediation also takes the outcome model into account while deriving the weights. Rather than being derived in a data adaptive manner, the weights for the TEQ score are based on a priori toxicologic information^75^. In the latter, the World Health Organization’s (WHO) well-established toxic equivalency factor (TEF) broadly incorporates additional biological information^76^. These scores are a measure of potency in reference to the chemical 2,3,7,8-tetrachlorodibenzo-*p*-dioxin. From these relative scores, we used the TEQ as created in Mitro et al. which represents a composite score of potency-weighted exposure^26^. The relevant contaminants with a TEQ score were weighted using these values, and each individual’s weighted average was used as a single mediator in the model. Contaminants without an available TEQ were excluded from the construction of the TEQ score.

#### Sensitivity analyses

We repeated the single mediator models with the inclusion of an exposure-mediator interaction term. In the multivariate setting, we also looked at the interaction between the PCA-based score and race. The purpose of this sensitivity analysis was to check if a potential interaction exists and to verify the mediation model mean structure assumptions. Next, we restricted the PCA summary score method to each subclass of POPs in our dataset (PCBs, non-ortho PCBs, non-dioxin-like PCBs, dioxins, furans). The purpose of this analysis was to understand which chemical classes most contribute to the global indirect effect. Lastly, we assessed the sensitivity of unmeasured confounding on our results using the mediation E-value for our PCA summary score method which can be calculated using the "EValue” R package or corresponding online calculator^77^.

## Results

We present descriptive statistics for all study variables in Table 1. In total, there were 930 White respondents and 321 Black respondents in our analytic sample. Of these, 53.3% of the White respondents and 54.8% of the Black respondents were female, and the Black respondents (Mean: 45.9; SD: 16.7) were younger than the White respondents (Mean: 52.5; SD: 19.8). Supplemental Fig. 2 shows the high collinearity among POPs both within and across chemical classes. The pairwise correlations between the PCBs range between 0.51 and 0.98. The dioxins and furans had pairwise correlations ranging from 0.33 to 0.80. The pairwise correlations between PCBs and dioxins/furans range from 0.25 to 0.72.

**Table 1:** NHANES sample characteristics, variables, and notation to be used in the analyses. Counts (%) and mean (SD) or median and interquartile range (IQR) are presented for the exposure and outcome variables.

Variable in conceptual model and notation	Variable	Total Sample	Sample Subset: White Respondents	Sample Subset: Black Respondents
Exposure *A*	Race	n = 1251	n = 930	n = 321
Outcome *Y*	LTL (T/S ratio)	1.011 (0.839, 1.175)	0.977 (0.824, 1.156)	1.067 (0.902, 1.267)
Mediators *M*_*1*_*,M*_*2*_*,…,M*_*9*_	PCBs	% Below LOD		
	PCB 74	30.50%		
	PCB 118	28.40%		
	PCB 126	21.70%		
	PCB 138	20.30%		
	PCB 153	16.90%		
	PCB 169	17.70%		
	PCB 170	26.40%		
	PCB 180	17.70%		
	PCB 187	32.10%		
*M*_*10*_*,M*_*11*_*,M*_*12*_	Dioxins	% Below LOD		
	DO3 (1,2,3,6,7,8-hxcdd)	20.90%		
	DO5 (1,2,3,4,6,7,8-hpcdd)	13.30%		
	D07 (1,2,3,4,6,7,8,9-ocdd)	20.50%		
*M*_*13*_*,M*_*14*_*,M*_*15*_	Furans	% Below LOD		
	F03 (2,3,4,7,8-pncdf)	36.90%		
	F04 (1,2,3,4,7,8-hxcdf)	27.30%		
	F08 (1,2,3,4,6,7,8-hxcdf)	25.30%		
Additional Adjustment Covariates *Z*_*1*_*, Z*_*2*_*,…Z*_*12*_	Survey cycle	‘99-’00 501	369	132
		‘01-’02 750	561	189
	Age	50.8 (19.3)	52.5 (19.8)	45.9 (16.7)
	Sex	M 579	434	145
		F 672	496	176
	Educational attainment	< 9th 81	57	24
		9–11 215	116	99
		HS/GED 324	254	70
		AA 338	255	83
		College + 293	248	45
	Serum cotinine (log-scale)	−0.55 (3.80)	−0.82 (3.81)	0.22 (3.69)
	Lipids (log-scale)	6.48 (0.22)	6.51 (0.22)	6.41 (0.19)
	White blood cell count	7.18 (2.15)	7.42 (2.14)	6.51 (2.03)
	% Lymphocytes	29.78 (9.07)	28.2 (8.20)	34.35 (9.90)
	% Neutrophils	58.43 (10.02)	60.01 (9.07)	53.70 (11.10)
	% Eosinophils	2.92 (2.44)	2.92 (2.07)	2.89 (3.28)
	% Basophils	0.67 (0.46)	0.69 (0.48)	0.61 (0.37)
	% Monocytes	8.25 (2.39)	8.17 (2.27)	8.47 (2.69)

**Figure 2:**
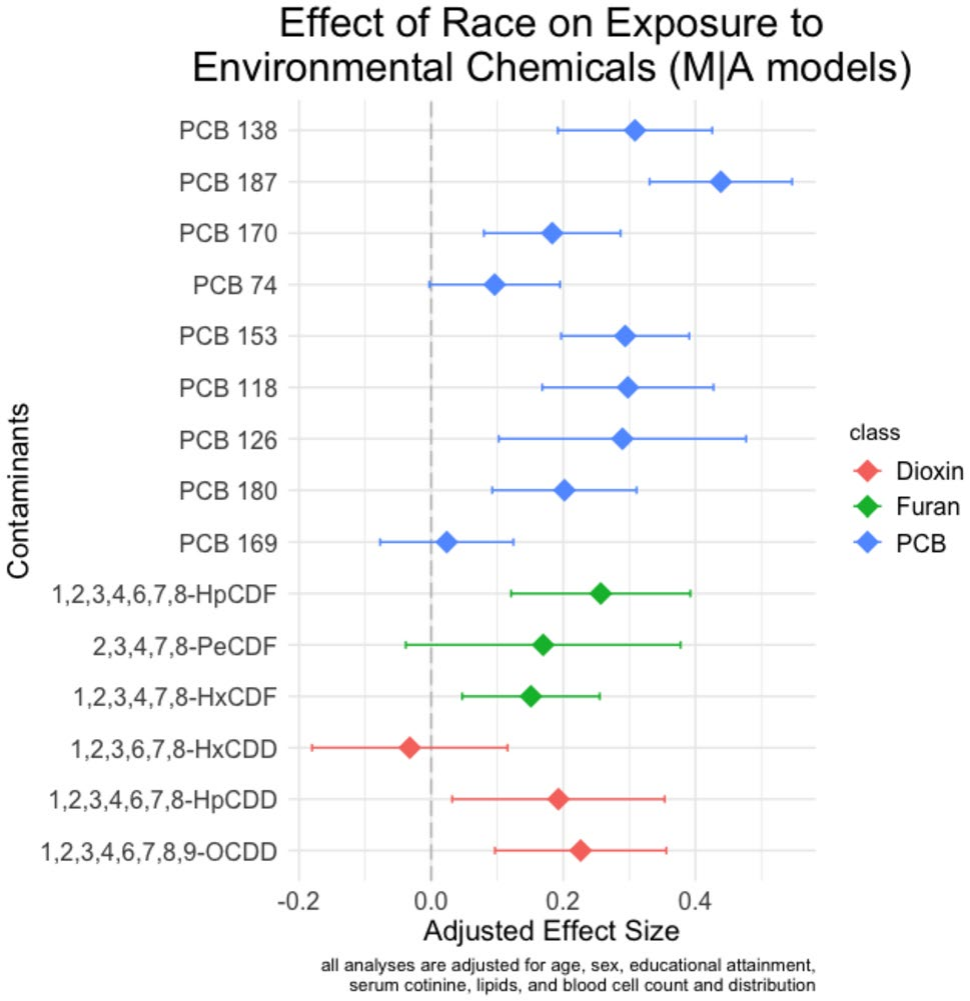
Results of the effect of self-reported Black race on exposure to environmental chemicals. Point estimates and 95% confidence intervals are shown.

**Figure 3:**
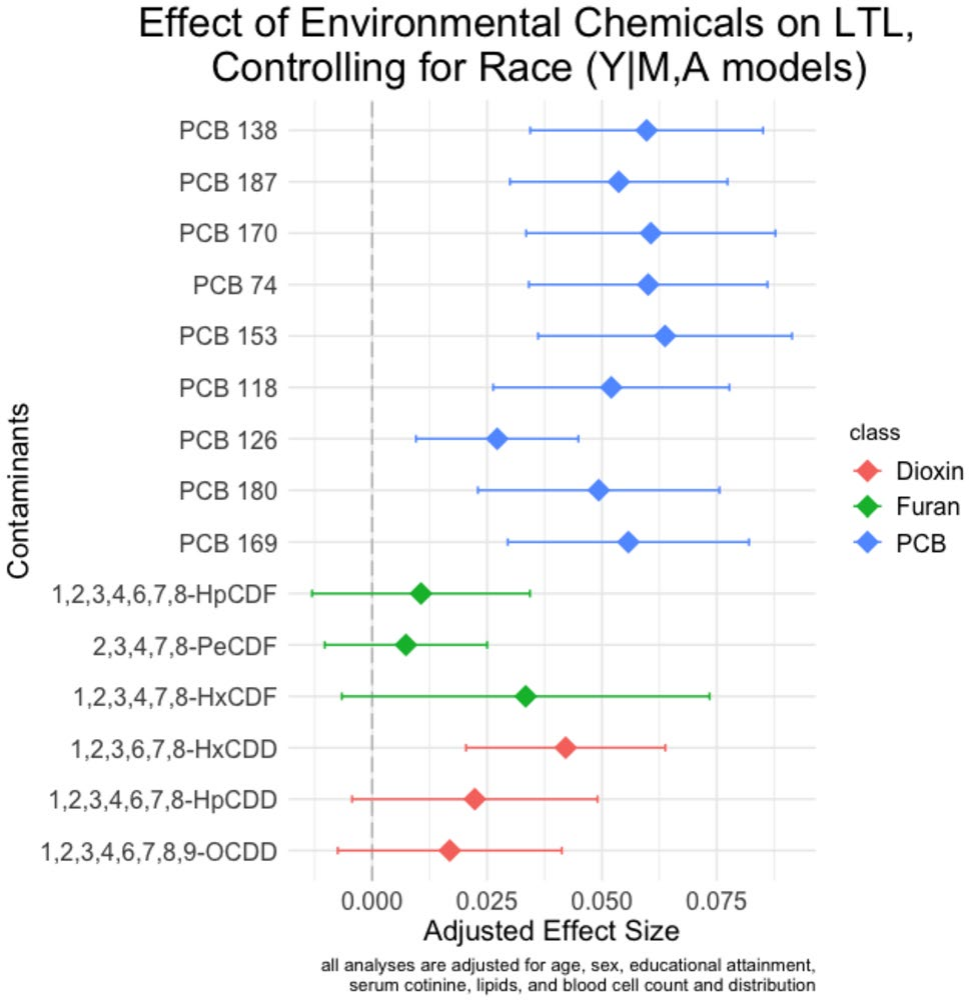
Results of the effect of environmental chemicals on leukocyte telomere length, controlling for self-reported race. Point estimates and 95% confidence intervals are shown.

To determine whether environmental chemicals mediate Black/White differences in LTL, we began by estimating the total effect of race on LTL. As shown in Supplemental Table 1, Black respondents had significantly longer LTL than White respondents in covariate-adjusted models (b = 0.054; CI 0.009, 0.099; p = 0.018). Next, we estimated indirect effects of the 15 potential mediators using the single mediator approach, with correction for multiple testing. In the *M|A,Z* models, Black respondents had significantly higher levels of 11 out of 15 environmental chemicals examined, including seven PCBs, two furans, and two dioxins (see Fig. 2). The chemicals that showed the greatest differences between racial groups were PCB 187, PCB 138, PCB 153, PCB 118, and PCB 126. In the *Y|M,A,Z* models, 10 of the 15 environmental chemicals were significantly associated with LTL (see Fig. 3). While each of the 10 PCBs were significantly associated with longer LTL, only one of the three dioxins was significantly associated with longer LTL, and none of the three furans were significantly associated with LTL.

**Figure 4:**
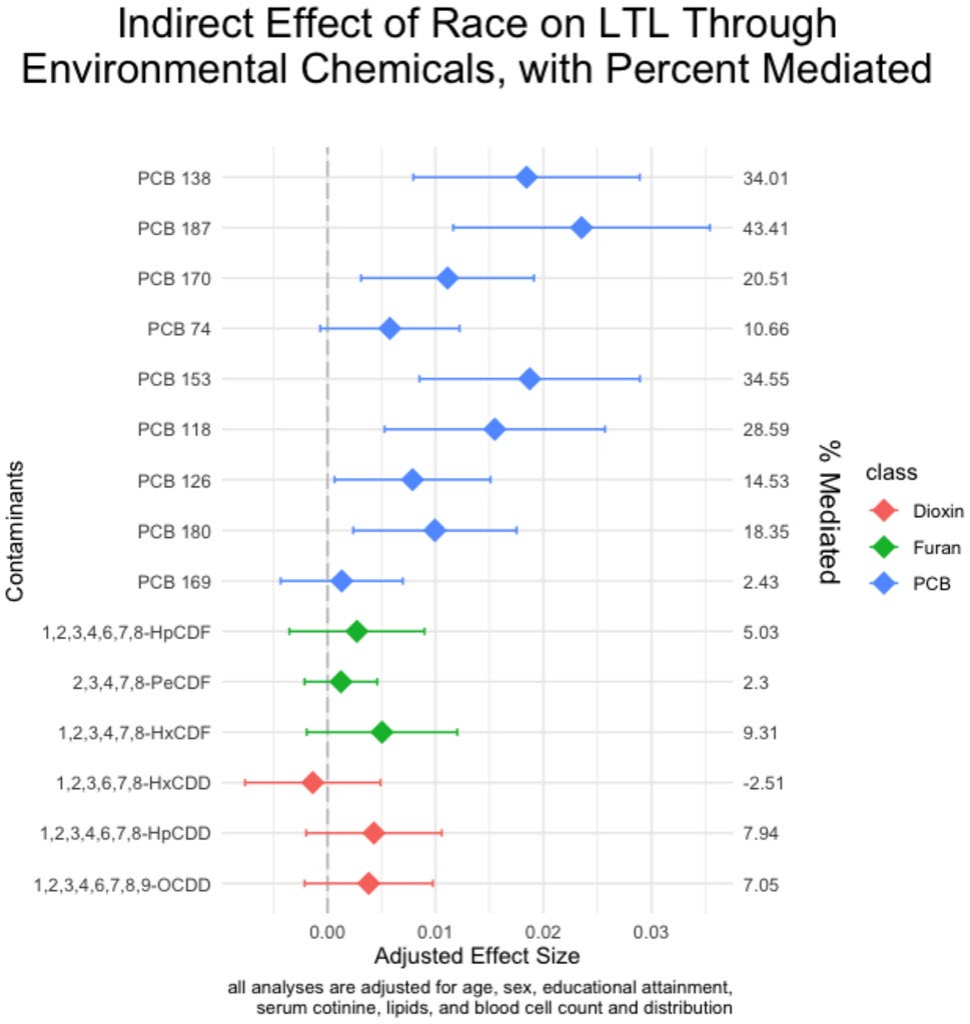
Results of the indirect effect of self-reported Black race on leukocyte telomere length through environmental chemicals. Point estimates and 95% confidence intervals are shown.

**Figure 5:**
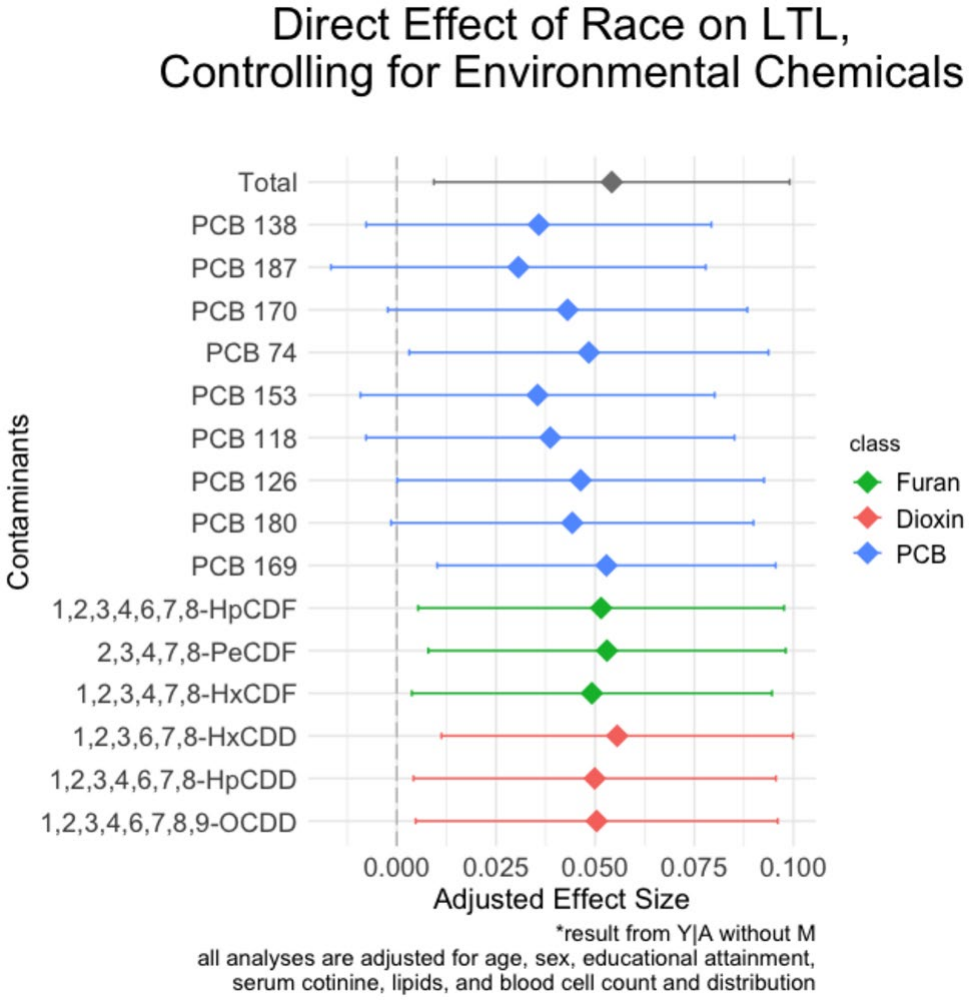
Results of the direct effect of self-reported Black race on leukocyte telomere length, controlling for environmental chemicals. Point estimates and 95% confidence intervals are shown.

Indirect effects are calculated from the *M|A,Z* and *Y|M,A,Z* models. The results for our mediation analysis, as shown in Fig. 4, showed that there were significant indirect effects (IE) of race on LTL through PCB 118 (IE = 0.015; 95% CI 0.005, 0.026; 28.6% mediated), PCB 138 (IE = 0.018; CI 0.008, 0.029; 34.0% mediated), PCB 153 (IE = 0.019; CI 0.009, 0.029; 34.5% mediated), PCB 170 (IE = 0.011; CI 0.003, 0.019; 20.5% mediated), PCB 180 (IE = 0.010; CI 0.002, 0.018; 18.4% mediated), and PCB 187 (IE = 0.024; CI 0.012, 0.035; 43.4% mediated) after correction for multiple testing. The direct effect of race on LTL was not statistically significant in models adjusting for PCBs that had significant indirect effects (see Fig. 5). We did not observe any significant interaction effects between the environmental chemical mediators and race from the sensitivity analysis (results not shown).

Given the limitations of the single mediator approach when examining a large number of highly collinear environmental chemicals as potential mediators, we also estimated indirect effects using multivariate and summary score mediator approaches. As shown in Tables 2 and 3, the unpenalized linear regression model estimated that 20.9% of the total effect of race on LTL was mediated by exposure to PCBs, furans, and dioxins (IE = 0.011; CI −0.004, 0.025), while the ridge penalized model estimated that 26.0% of the total effect was mediated by the full set of 15 environmental chemicals (IE = 0.013; CI 0.001, 0.023).

**Table 2:** Overview of analytical mediation models and methods considered in this analysis.

Method	Brief Description	Accounts for Multiple Mediators	Accounts for complex sample design	Summary of Results
Pairwise Mediation	Examines one mediator at a time	No, each mediator is modeled independently from the others	Yes	Significant indirect effects observed for six PCBs
Unpenalized Linear Regression	Outcome model is a multivariable linear regression model (one outcome) and mediator model is a multivariate linear regression model (multiple outcomes)	Yes, although there are issues with variance inflation due to highly collinear POPs	No	Non-significant global mediation effect observed. Estimated global mediation effect of 20.9%
Ridge Penalized Linear Regression	Same as the unpenalized linear regression approach, with the exception that the outcome model now has a ridge penalty term. The ridge penalty shrinks regression coefficients toward zero in an effort to reduce the impact of variance inflation	Yes, shrinkage/penalization on the effects of the mediators in the outcome model reduce variance inflation caused by collinearity	No	Significant global mediation effect observed. Estimated global mediation effect of 26.0%
PCA Score	Summary score method: calculate the first principal component and use the loadings as weights to compute a summary exposure score. Take the summary exposure score and run it through the single mediator framework	Yes, the summary score is derived using all POPs	Yes	Significant global mediation effect observed. Estimated global mediation effect of 34.8%
PDM Score	Summary score method: PDM is similar to PCA, with the difference being that the first principal direction of mediation also takes the outcome model into account while deriving the loadings. Take the loadings for the first principal direction of mediation to compute a summary exposure score. Take the summary exposure score and run it through the single mediator framework	Yes, the summary score is derived using all POPs. However, because the PDM formulation includes an unpenalized outcome model, there are likely potential issues with variance inflation due to collinearity	Yes	No global mediation effect observed. Estimated global mediation effect of 0.6%
TEQ Score	Summary score method: The World Health Organization’s (WHO) toxic equivalency factor (TEF) scores are used as weights to construct a summary exposure score. Take the summary exposure score and run it through the single mediator framework	Yes, the summary score is derived using all POPs	Yes	Significant global mediation effect observed. Estimated global mediation effect of 28.8%

**Table 3:** Indirect effect and direct effect summary. Presented results include the indirect effect, direct effect, percent indirect effect, and corresponding confidence interval and p-values for the indirect effects across different mediation models.

Model	IDE	DE	% IDE	95% CI IDE	P-Value IDE
PCB 74	0.006	0.048	10.7%	(−0.001, 0.012)	0.148
PCB 118	0.015	0.039	28.6%	(0.005, 0.026)	0.011
PCB 126	0.008	0.046	21.7%	(0.001, 0.015)	0.070
PCB 138	0.018	0.036	34.0%	(0.008, 0.029)	0.003
PCB 153	0.019	0.035	34.5%	(0.009, 0.029)	0.002
PCB 169	0.001	0.053	2.4%	(−0.004, 0.007)	0.671
PCB 170	0.011	0.043	20.5%	(0.003, 0.019)	0.019
PCB 180	0.010	0.044	18.4%	(0.002, 0.018)	0.025
PCB 187	0.024	0.031	43.4%	(0.012, 0.035)	0.002
D03	0.001	0.053	2.3%	(−0.002, 0.005)	0.540
D05	0.004	0.050	7.9%	(−0.002, 0.011)	0.269
D07	0.004	0.050	7.0%	(−0.002, 0.010)	0.283
F03	−0.001	0.056	−2.5%	(−0.008, 0.005)	0.671
F04	0.005	0.049	9.3%	(−0.002, 0.012)	0.260
F08	0.003	0.052	5.0%	(−0.004, 0.009)	0.492
Unpenalized Linear Regression	0.011	0.040	20.9%	(−0.004, 0.025)	0.136
Ridge Regression	0.013	0.037	26.0%	(0.001, 0.023)	0.034
PCA: All Toxicants	0.019	0.035	34.8%	(0.009, 0.029)	0.001
TEQ	0.016	0.039	28.8%	(0.005, 0.026)	0.003
PDM	0.000	0.054	0.6%	(−0.001, 0.002)	0.692

P-values in the single mediator models are corrected for multiple testing using the Benjamini–Hochberg correction.

Next, we examined three summary score methods (see Table 4 for a list of weights corresponding to each summary score). Since all of the weights for the PCA-based and TEQ-based exposure scores were non-negative, we can then conclude that higher values represent higher cumulative POP exposure. However, because the weights for the PDM-based exposure score are in both positive and negative directions, the interpretation of the exposure score is less straightforward. PCA (IE = 0.019; CI 0.009, 0.029; p = 0.001; 34.8% mediated) and TEQ (IE = 0.016; CI 0.005, 0.026; p = 0.003; 28.8% mediated) showed significant indirect effects, while PDM showed a non-significant indirect effect (IE = 0.000; CI −0.001, 0.002; 0.6% mediated). In the PCA score model, the inclusion of an interaction term between race and the PCA-based score in the outcome model did not result in a better model fit compared to an outcome model without the race by PCA score interaction term (p = 0.980). In sensitivity analyses, we examined the PCA approach within environmental chemical subclasses (see Table 4 for weights). We found a significant indirect effect of PCBs (IE = 0.020; CI 0.010, 0.029; p = 0.001; 36.2% mediated) but no significant indirect effects of furans or dioxins (see Table 5). Furthermore, we subdivided the PCBs into non-ortho-PCBs (IE = 0.008; CI 0.001, 0.015; p = 0.025; 14.4% mediated) and non-dioxin-like PCBs (IE = 0.018; CI 0.009, 0.028; p = 0.001; 25.9% mediated), which revealed that both subclasses were associated with a significant mediation effect.

**Table 4:** Exposure score weights for different analytic models considered.

Mediation model	PCA (all)	PDM (all)	TEQ (all)	PCA (PCB)	PCA (dioxin)	PCA (furan)	PCA (non-dioxin-like)	PCA (non-ortho-like)
PCB 74	0.285	−0.196	–	−0.333	–	–	−0.377	–
PCB 118	0.283	0.067	0.030	−0.330	–	–	–	
PCB 126	0.224	0.071	0.100	−0.242	–	–	–	−0.707
PCB 138	0.294	0.385	–	−0.352	–	–	−0.410	–
PCB 153	0.298	0.040	–	−0.360	–	–	−0.421	-
PCB 169	0.275	−0.107	0.030	−0.317	–	–	–	−0.707
PCB 170	0.292	0.335	–	−0.352	–	–	−0.416	–
PCB 180	0.286	−0.740	–	−0.347	–	–	−0.410	–
PCB 187	0.290	−0.147	–	−0.351	–	–	−0.413	–
D03	0.213	−0.053	0.100	–	0.520	–	–	–
D05	0.216	−0.132	0.010	–	0.595	–	–	–
D07	0.232	−0.127	0.0003	–	0.613	–	–	–
F03	0.243	0.042	0.300	–	–	−0.577	–	–
F04	0.246	−0.093	0.100	–	–	−0.638	–	–
F08	0.145	0.249	0.010	–	–	−0.510	–	–

**Table 5:** PCA subclass sensitivity analysis and results including direct, indirect effect, percent indirect effect, and confidence intervals and p-values for each subclass mediation model.

Mediation Model	Indirect Effect of Race on LTL	Direct Effect	% Indirect Effect	Confidence Interval for IE	P-value for IE
PCA: Non-ortho-like PCBs	0.008	0.046	14.4	(0.001, 0.015)	0.025
PCA: Non-dioxin-like PCBs	0.018	0.036	25.9	(0.009, 0.028)	0.001
PCA: All PCBs	0.020	0.035	36.2	(0.010, 0.029)	0.001
PCA: Dioxins	0.005	0.050	8.6	(−0.002, 0.011)	0.166
PCA: Furans	0.006	0.048	1.06	(0.000, 0.012)	0.069

Our sensitivity analysis of the PCA mediation model included a calculation of the E-value, which estimates the strength of association that would be needed with unmeasured confounders to attenuate the mediation effect to a null level. Based on our estimates, the E-value was 1.35 (lower bound 1.22), meaning an unmeasured confounder would need to have a risk ratio of 1.35 in association with LTL and the summary score of environmental chemicals from PCA to diminish the indirect effect that we estimated down to zero.

## Discussion

The purpose of this study was to determine whether exposure to POPs helps explain why Black Americans have longer LTL than White Americans, despite having more risk factors for short LTL^1^. Previous research using nationally representative NHANES data has shown that Black Americans are exposed to higher levels of POPs than White Americans^44^. While differences are not explained by behavioral factors, such as diet or smoking^30,44^, a growing body of evidence suggests social structural factors like residential^48,49^ and occupational^52,53^ segregation are important contributors to racial disparities in exposure to environmental chemicals^78^. Given recent observational and experimental studies linking exposure to POPs with longer LTL^26,27–33,36^, we hypothesized that Black/White differences in LTL are explained by differences in exposure to PCBs, furans, and dioxins. Using various analytic methods, we found support for the hypothesis that exposure to POPs, and PCBs in particular, partially mediates Black/While differences in LTL.

Although previous research has established that Black Americans have higher exposure to POPs^35,44–46^ and that exposure to POPs is associated with longer LTL^26,27–33^, no prior studies have considered the extent to which differences in exposure to environmental chemicals contribute to race differences in LTL. In single-pollutant models, we found significant IEs of race on LTL through five non-dioxin-like PCBs (138, 153, 170, 180, and 187) and one that is not dioxin-like, PCB 118. The estimated percent mediated ranged from 18 to 43%. Given the highly correlated nature of environmental chemicals, we also explored multivariate and summary score mediator approaches. We found evidence of significant IEs in ridge regression and models examining summative exposure scores (from PCA and the TEQ score). The estimated percent mediated ranged from 26 to 35% using these approaches. Sensitivity analyses examining principal components within chemical subclasses revealed significant IEs of PCBs (both non-ortho PCBs and non-dioxin-like PCBs) but not furans or dioxins. These findings suggest that exposure to PCBs is a potentially modifiable mechanism underlying Black/White differences in LTL in the US. Given evidence of causal associations between longer telomere length and some types of cancer^13–21^, it is important to identify modifiable risk factors for long LTL.

Although the various statistical approaches used in this study produced similar results overall, we observed some potentially important differences across methods. Of the multivariate and summary score mediation approaches, four out of the five methods estimated a percent mediated of greater than 20%, indicating a general agreement across methods. That being said, we also observed that multivariate mediation approaches that do not directly address the impact of variance inflation due to correlated POPs (i.e., unpenalized linear regression and PDM) show either no global mediation effect or an attenuated global mediation effect compared to other methods. As a result, OLS-based approaches should be used with caution when jointly modeling highly collinear environmental chemicals. Alternatively, when considering a joint analysis on the mediation effect attributable to exposure mixtures, we recommend either penalizing the regression coefficients (through a ridge, adaptive elastic net, or sparse group lasso penalty, for example), or collapsing exposures into summary scores with the weights selected in a data adaptive way (as with principal components analysis). We did not consider high-dimensional multivariate mediation methods^79–82^ because we only had 15 mediators in our analysis.

### Strengths, limitations, and directions for future research

This study has several strengths, including the data source and analysis methods. First, the analysis was conducted on nationally representative data, which minimizes selection bias and helps ensure that results are generalizable to Black and White adults living in the US. Historically, much research regarding differences in LTL has been based on smaller samples with less sample diversity. NHANES is one of few studies with data on LTL and POPs for Black and White adults. The Anniston, Alabama sample is another, but it includes a smaller, more highly exposed population^29^. Another strength of our analysis is the methodological rigor of our mediation analysis. While not all existing mediation models could incorporate survey weights and sampling design elements, we have clearly laid out which methods have the capability to account for the weighting, strata, and PSU variables. It is possible that generalizability and correct inference are more limited when these variables cannot be included. Despite each method having limitations, the concordant results across methods with and without survey design elements strengthen our overall conclusions.

Despite its strengths, this study has several limitations. First, NHANES is a cross-sectional study, which means that we only have measures of POPs and LTL at one point in time. Given the long half-life of POPs in blood, it is possible that our measurements represent chronic exposure. However, these measures may not capture chemical exposures in early life, which may be an etiologically relevant time period. Studies with longitudinal data—including studies with children and adolescents—are needed to determine whether change in exposure to POPs is associated with change in LTL.

Additional limitations related to our exposure data include an inability to account for exposure measurement error at the statistical modeling stage and detection limit issues, which substantially reduce the set of exposures that can be used in the imputation and mediation models. Here we measured POPs, but as a future direction, it is important to include other chemicals (e.g., metals) that have previously been associated with LTL^83^ and race/ethnicity^84,85^.

Other potential limitations of this study relate to the mediation analyses. Mediation methods have been proposed under the traditional causal steps approach^67^ and the potential outcomes formulation^71,72^. To interpret the results of mediation analysis as causal effects, a number of assumptions must be met. Although we explored the possibility of exposure-mediator interaction, accounted for measured confounders of all relationships, and assessed the robustness of our results using the E-value, failure to account for unmeasured confounders could lead to non-identifiability of causal mediation effects in our observed data. For example, genetic factors that are causally related to telomere length may also be associated with self-reported race. A recent study using data from the NHLBI Trans-Omics for Precision Medicine (TOPMed) program identified 59 genetic variants associated with telomere length (estimated from whole-genome sequences) in an ancestrally diverse sample including people of European and African ancestry^86^. There was little evidence of effect size heterogeneity across ancestry groups, but the frequency of the variants differed for Black and White participants, suggesting that some of the observed Black/White differences in telomere length may be due to genetic factors. Unfortunately, NHANES does not have all of the genetic data needed to construct the TOPMed telomere length polygenic trait score. Thus, we cannot rule out the possibility of unmeasured confounding due to genetic determinants of telomere length.

In addition to the possibility of unmeasured confounding by genetic factors, our models may have violated the assumption that no confounders of mediator-outcome associations were affected by the exposure. Given that self-reported race is an important determinant of educational attainment in the US context, controlling for education as a potential confounder of associations between POPs and LTL risks violating one of the assumptions of causal mediation.

A causal interpretation also requires correctly specifying the temporal order of the variables. Here race precedes POP exposure, and it is unlikely that LTL would impact serum chemical concentrations. Thus, we are confident that the temporal order is correctly specified, despite the use of cross-sectional data. A final potential limitation of the mediation analysis concerns our exposure variable. Some causal inference scholars have argued that race coefficients cannot be interpreted causally because there is no reasonable hypothetical intervention on race^87–89^. However, this argument is not universally accepted. Because race is a social construction rather than a biological category, a number of scholars reject the claim that race is not manipulable^90–92^. The meaning of race is culturally defined^90^, and both race classifications and race relations vary across place and time^91,92^. Others have called into question the assumption that causal claims are only reasonable in the context of well-defined interventions^90,91,93^. Although we believe that the use of self-reported race is justified in this analysis, we also recognize the importance of expanding our model to include more proximate causes of telomere length that are amenable to standard policy interventions^94^.

### Conclusions

Black/White differences in LTL are partially explained by differences in exposure to POPs, a potentially modifiable environmental risk factor. More work is needed to understand the complex relationships between environmental racism, chemical exposures, LTL, and cancer disparities. Although our results suggest that mediation analysis is a useful technique for identifying environmental mechanisms underlying race differences in LTL, differences across methods arising from the way in which variance inflation is handled suggest that OLS-based approaches should be used with caution for correlated exposure data. Researchers who wish to study environmental chemicals as mediators should explore appropriate analytic tools for their research question, including considerations about the collinear nature of their data and the capability of various methods to account for survey design elements when necessary.

## Supplementary Information

Supplementary Information.

## Data Availability

The datasets analyzed during the current study are available from the NHANES Study, https://wwwn.cdc.gov/nchs/nhanes/.

## References

[1] Needham BL (2019). Do black/white differences in telomere length depend on socioeconomic status?. Biodemogr. Soc. Biol..

[2] Benetos A, Aviv A (2017). Ancestry, telomere length, and atherosclerosis risk. Circ. Cardiovasc. Genet..

[3] Hamad R, Walter S, Rehkopf DH (2016). Telomere length and health outcomes: A two-sample genetic instrumental variables analysis. Exp. Gerontol..

[4] Hansen ME (2016). Shorter telomere length in Europeans than in Africans due to polygenetic adaptation. Hum. Mol. Genet..

[5] Blackburn EH, Epel ES, Lin J (2015). Human telomere biology: a contributory and interactive factor in aging, disease risks, and protection. Science.

[6] Harley CB, Futcher AB, Greider CW (1990). Telomeres shorten during ageing of human fibroblasts. Nature.

[7] von Zglinicki T (2002). Oxidative stress shortens telomeres. Trends Biochem. Sci..

[8] Blackburn EH (1997). The telomere and telomerase: Nucleic acid-protein complexes acting in a telomere homeostasis system: A review. Biochem.-N.Y.-Eng. Transl. Biokhimiya.

[9] Blackburn EH (2000). Telomere states and cell fates. Nature.

[10] Blasco MA (2005). Telomeres and human disease: Ageing, cancer and beyond. Nat. Rev. Genet..

[11] Kennedy BK (2014). Geroscience: Linking aging to chronic disease. Cell.

[12] López-Otín C, Blasco MA, Partridge L, Serrano M, Kroemer G (2013). The hallmarks of aging. Cell.

[13] Codd V (2013). Identification of seven loci affecting mean telomere length and their association with disease. Nat. Genet..

[14] Gao K (2019). Exploring the causal pathway from telomere length to Alzheimer’s disease: an update Mendelian randomization study. Front. Psych..

[15] Gao Y (2020). Assessing the relationship between leukocyte telomere length and cancer risk/mortality in UK biobank and TCGA datasets with the genetic risk score and Mendelian randomization approaches. Front. Genet..

[16] Haycock PC (2017). Association between telomere length and risk of cancer and non-neoplastic diseases: A Mendelian randomization study. JAMA Oncol..

[17] Kachuri L (2019). Mendelian randomization and mediation analysis of leukocyte telomere length and risk of lung and head and neck cancers. Int. J. Epidemiol..

[18] Kuo CL, Pilling LC, Kuchel GA, Ferrucci L, Melzer D (2019). Telomere length and aging-related outcomes in humans: A Mendelian randomization study in 261,000 older participants. Aging Cell.

[19] Zhang C (2015). Genetic determinants of telomere length and risk of common cancers: a Mendelian randomization study. Hum. Mol. Genet..

[20] Zhang X (2017). The association of telomere length in peripheral blood cells with cancer risk: A systematic review and meta-analysis of prospective studies. Cancer Epidemiology and Prevention Biomarkers.

[21] The Telomeres Mendelian Randomization Collaboration, et al. Association between telomere length and risk of cancer and non-neoplastic diseases: A mendelian randomization Study. *JAMA Oncol.* **3(5):** 636–651 (2017). 10.1001/jamaoncol.2016.5945 PMC5638008 28241208

[22] IARC (International Agency for Research on Cancer). Chemical agents and related occupations. IARC Monographs on the Evaluation of Carcinogenic Risks to Humans. 339–378. (2012). PMC4781612 23189753

[23] EPA. *Persistent Organic Pollutants: A Global Issue, A Global Response*. EPA. Accessed October 12, 2022. [https://www.epa.gov/international-cooperation/persistent-organic-pollutants-global-issue-global-response#pops] (2022).

[24] Lauby-Secretan B (2013). Carcinogenicity of polychlorinated biphenyls and polybrominated biphenyls. Lancet Oncol..

[25] Scinicariello F, Buser MC (2015). Polychlorinated biphenyls and leukocyte telomere length: an analysis of NHANES 1999–2002. EBioMedicine.

[26] Mitro SD, Birnbaum LS, Needham BL, Zota AR (2016). Cross-sectional associations between exposure to persistent organic pollutants and leukocyte telomere length among US adults in NHANES, 2001–2002. Environ. Health Perspect..

[27] Gibson EA (2019). An overview of methods to address distinct research questions on environmental mixtures: An application to persistent organic pollutants and leukocyte telomere length. Environ. Health.

[28] Patel CJ, Manrai AK, Corona E, Kohane IS (2017). Systematic correlation of environmental exposure and physiological and self-reported behaviour factors with leukocyte telomere length. Int. J. Epidemiol..

[29] Callahan CL (2017). Serum polychlorinated biphenyls and leukocyte telomere length in a highly-exposed population: The Anniston Community Health Survey. Environ. Int..

[30] Pavuk M (2014). Serum concentrations of polychlorinated biphenyls (PCBs) in participants of the Anniston Community Health Survey. Sci. Total Environ..

[31] Shin JY, Choi YY, Jeon HS, Hwang JH, Kim SA (2010). Low-dose persistent organic pollutants increased telomere length in peripheral leukocytes of healthy Koreans. Mutagenesis.

[32] Karimi B, Nabizadeh R, Yunesian M (2020). Association between leukocyte telomere length and serum concentrations of PCBs and organochlorine pesticides. Arch. Environ. Contam. Toxicol..

[33] Karimi B, Nodehi RN, Yunesian M (2020). Serum level of PCBs and OCPs and leukocyte telomere length among adults in Tehran, Iran. Chemosphere.

[34] Ziegler S (2017). Accelerated telomere shortening in peripheral blood lymphocytes after occupational polychlorinated biphenyls exposure. Arch. Toxicol..

[35] Guzzardi MA (2016). Exposure to persistent organic pollutants predicts telomere length in older age: results from the Helsinki birth cohort study. Aging Dis..

[36] VanEtten SL (2020). Telomeres as targets for the toxicity of 2, 3, 7, 8-tetrachlorodibenzo-p-dioxin (TCDD) and polychlorinated biphenyls (PCBs) in rats. Toxicol. Appl. Pharmacol..

[37] Ruden M, Puri N (2013). Novel anticancer therapeutics targeting telomerase. Cancer Treat. Rev..

[38] Ghosh S, De S, Dutta SK (2007). Altered Protein Expressions in Chronic PCB-153–Induced Human Liver (HepG2) Cells. Int. J. Toxicol..

[39] Casati LGMSS, Catalani IZMDP, Marafante E (1998). Modulation of proto-oncogene expression by polychlorinated biphenyls in 3T3-L1 cell line. J. Toxicol. Environ. Health A.

[40] Daniel M, Peek GW, Tollefsbol TO (2012). Regulation of the human catalytic subunit of telomerase (hTERT). Gene.

[41] Sarkar P, Shiizaki K, Yonemoto J, Sone H (2006). Activation of telomerase in BeWo cells by estrogen and 2,3,7,8-tetrachlorodibenzo-p-dioxin in co-operation with c-Myc. Int. J. Oncol..

[42] Chopra M, Schrenk D (2011). Dioxin toxicity, aryl hydrocarbon receptor signaling, and apoptosis-persistent pollutants affect programmed cell death. Crit. Rev. Toxicol..

[43] ATSDR. Toxicological Profile for Polychlorinated Biphenyls (PCBs). Atlanta, GA:Agency for Toxic Substances and Disease Registry. [accessed 18 February 2021][ http://www.atsdr.cdc.gov/toxprofiles/tp17.pdf]. (2000). 36888731

[44] Xue J, Liu SV, Zartarian VG, Geller AM, Schultz BD (2014). Analysis of NHANES measured blood PCBs in the general US population and application of SHEDS model to identify key exposure factors. J. Eposure Sci. Environ. Epidemiol..

[45] Yang E (2018). Exposure of dioxin-like chemicals in participants of the Anniston community health survey follow-up. Sci. Total Environ..

[46] Jones RR (2021). Residential proximity to dioxin-emitting facilities and risk of non-hodgkin lymphoma in the NIH-AARP diet and health study. Cancer Epidemiol. Biomark. Prev..

[47] Jargowsky PA (2018). The persistence of segregation in the 21st century. Law Ineq..

[48] Mohai P, Pellow D, Roberts JT (2009). Environmental justice. Annu. Rev. Environ. Resour..

[49] Taylor D (2014). Toxic Communities: Environmental Racism, Industrial Pollution, and Residential Mobility.

[50] Reardon SF, Fox L, Townsend J (2015). Neighborhood income composition by household race and income, 1990–2009. Ann. Am. Acad. Pol. Soc. Sci..

[51] Williams DR, Mohammed SA, Leavell J, Collins C (2010). Race, socioeconomic status and health: Complexities, ongoing challenges and research opportunities. Ann. N. Y. Acad. Sci..

[52] Angelon-Gaetz KA, Richardson DB, Wing S (2010). Inequalities in the nuclear age: impact of race and gender on radiation exposure at the Savannah River Site (1951–1999). NEW Solut.: J. Environ. Occupat. Health Policy.

[53] Briggs NC (2003). Occupational risk factors for selected cancers among African American and White men in the United States. Am. J. Public Health.

[54] Weintraub M, Birnbaum LS (2008). Catfish consumption as a contributor to elevated PCB levels in a non-Hispanic black subpopulation. Environ. Res..

[55] Centers for Disease Control and Prevention (CDC). National Center for Health Statistics (NCHS). National Health and Nutrition Examination Survey Questionnaire (or Examination Protocol, or Laboratory Protocol). Hyattsville, MD: U.S. Department of Health and Human Services, Centers for Disease Control and Prevention, [accessed February 18 2021] [https://wwwn.cdc.gov/nchs/data/nhanes/1999-2000/manuals/1999-2000_MEC_Laboratory_Manual.pdf]. (1999).

[56] Cawthon RM (2002). Telomere measurement by quantitative PCR. Nucl. Acids Res..

[57] Lin J (2010). Analyses and comparisons of telomerase activity and telomere length in human T and B cells: insights for epidemiology of telomere maintenance. J. Immunol. Methods.

[58] Centers for Disease Control and Prevention (CDC). *NHANES 1999–2000: Telomere mean and standard deviation (surplus) data documentation, codebook, and frequencies*. Centers for Disease Control and Prevention. [accessed February 18, 2021][https://wwwn.cdc.gov/Nchs/Nhanes/1999-2000/TELO_A.htm]. (2015).

[59] Needham BL (2013). Socioeconomic status, health behavior, and leukocyte telomere length in the National Health and Nutrition Examination Survey, 1999–2002. Soc. Sci. Med..

[60] Jones CP (2000). Levels of racism: a theoretic framework and a gardener's tale. Am. J. Public Health.

[61] CDC (Centers for Disease Control and Prevention). Laboratory Procedure Manual: PCBs and Persistent Pesticides in Serum. 2001–2002 ed. Atlanta, GA:CDC, National Center for Environmental Health. Available: http://www.cdc.gov/nchs/ data/nhanes/nhanes_01_02/l28poc_b_met_pcb_ pesticides.pdf [accessed 18 February 2021]. (2002a).

[62] CDC (Centers for Disease Control and Prevention). Laboratory Procedure Manual: PCDDs, PCDFs, and cPCBs in Serum. 2001–2002 ed. Atlanta, GA:National Center for Environmental Health. [accessed 18 February 2021][http://www.cdc.gov/nchs/data/nhanes/nhanes_01_02/l28poc_b_met_dioxin_pcb]. (2022b)

[63] Codru, N. *et al.* Diabetes in relation to serum levels of polychlorinated biphenyls and chlorinated pesticides in adult Native Americans. *Environ. Health Perspect.* **115**(10), 1442–1447 (2007). 10.1289/ehp.10315 PMC2022671 17938733

[64] Schisterman EF, Whitcomb BW, Buck Louis GM, Louis TA (2005). Lipid adjustment in the analysis of environmental contaminants and human health risks. Environ. Health Perspect..

[65] Boss J (2019). Estimating outcome-exposure associations when exposure biomarker detection limits vary across batches. Epidemiology.

[66] Lubin JH (2004). Epidemiologic evaluation of measurement data in the presence of detection limits. Environ. Health Perspect..

[67] Baron RM, Kenny DA (1986). The moderator–mediator variable distinction in social psychological research: Conceptual, strategic, and statistical considerations. J. Pers. Soc. Psychol..

[68] Sobel ME (1982). Asymptotic confidence intervals for indirect effects in structural equation models. Sociol. Methodol..

[69] Benjamini Y, Hochberg Y (1995). Controlling the false discovery rate: a practical and powerful approach to multiple testing. J. Roy. Stat. Soc. B.

[70] Lumley, T. survey: analysis of complex survey samples. R package version 4.0. (2020).

[71] Robins, J. M., & Greenland, S. Identifiability and exchangeability for direct and indirect effects. *Epidemiology*, 143–155. (1992). 10.1097/00001648-199203000-00013 1576220

[72] Pearl, J. Direct and indirect effects. In J. Breese & D. Koller (Eds.) *Proceedings of the 17th Conference on Uncertainty in Artificial Intelligence.* San Francisco, CA: Morgan Kaufmann. 411–420, (2001).

[73] Hoerl AE, Kennard RW (1970). Ridge regression: Biased estimation for nonorthogonal problems. Technometrics.

[74] Chén OY (2018). High-dimensional multivariate mediation with application to neuroimaging data. Biostatistics.

[75] Van den Berg M (2006). The 2005 World Health Organization reevaluation of human and mammalian toxic equivalency factors for dioxins and dioxin-like compounds. Toxicol. Sci..

[76] *World Health Organization*. Dioxins and their effects on human health. www.who.int/news-room/fact-sheets/detail/dioxins-and-their-effects-on-human-health.

[77] VanderWeele TJ, Ding P (2017). Sensitivity analysis in observational research: introducing the E-value. Ann. Intern. Med..

[78] Lane, H. M., Morello-Frosch, R., Marshall, J. D., & Apte, J. S. Historical redlining is associated with present-day air pollution disparities in US cities. *Environmental Science & Technology Letters*. (2022). 10.1021/acs.estlett.1c01012 PMC9009174 35434171

[79] Song Y (2020). Bayesian shrinkage estimation of high dimensional causal mediation effects in omics studies. Biometrics.

[80] Zhang H, Zheng Y, Hou L, Zheng C, Liu L (2021). Mediation analysis for survival data with high-dimensional mediators. Bioinformatics.

[81] Zhang H (2016). Estimating and testing high-dimensional mediation dffects in dpigenetic studies. Bioinformatics.

[82] Zhao, Y., & Luo, X. Pathway lasso: estimate and select sparse mediation pathways with high dimensional mediators. *arXiv preprint *arXiv:1603.07749. (2016).

[83] Zota AR (2015). Associations of cadmium and lead exposure with leukocyte telomere length: findings from National Health and Nutrition Examination Survey, 1999–2002. Am. J. Epidemiol..

[84] Cassidy-Bushrow AE (2017). Burden of higher lead exposure in African-Americans starts in utero and persists into childhood. Environ. Int..

[85] Nguyen VK (2020). A comprehensive analysis of racial disparities in chemical biomarker concentrations in United States women, 1999–2014. Environ. Int..

[86] Taub MA (2022). Genetic determinants of telomere length from 109,122 ancestrally diverse whole-genome sequences in TOPMed. Cell Genomics.

[87] Hernán MA (2017). Does water kill? A call for less casual causal inferences. Ann. Epidemiol..

[88] Holland PW (1986). Statistics and causal inference. J. Am. Stat. Assoc..

[89] VanderWeele, T. J., & Robinson, W. R. On causal interpretation of race in regressions adjusting for confounding and mediating variables. *Epidemiol. (Cambridge, Mass)*, **25(4),** 473. (2014). 10.1097/EDE.0000000000000105 PMC4125322 24887159

[90] Glymour C, Glymour MR (2014). Commentary: race and sex are causes. Epidemiology.

[91] Glymour, M. M., & Spiegelman, D. Evaluating public health interventions: 5. Causal inference in public health research—do sex, race, and biological factors cause health outcomes? *Am. J. Public Health* **107**(1), 81–85 (2017). 10.2105/AJPH.2016.303539 PMC5308179 27854526

[92] Krieger N (2014). On the Causal Interpretation of Race. Epidemiology.

[93] Schwartz, S., Prins, S. J., Campbell, U. B., & Gatto, N. M. Is the “well-defined intervention assumption” politically conservative? *Soc. Sci. Med. (1982)*, **166,** 254. (2016). 10.1016/j.socscimed.2015.10.054 PMC4848169 26777446

[94] Bellavia, A., Zota, A. R., Valeri, L., & James-Todd, T. Multiple mediators approach to study environmental chemicals as determinants of health disparities. *Environ. Epidemiol. (Philadelphia, Pa.)*, ***2*****(2),** 1 (2018). 10.1097/EE9.0000000000000015 PMC6748334 31531412

