# Supplementary material for: Persistent organic pollutant exposure contributes to Black/White differences in leukocyte telomere length in the National Health and Nutrition Examination Survey
Source: Sci Rep. 2022 Nov 19;12:19960. doi: 10.1038/s41598-022-24316-0 (PMC9675834; doi:10.1038/s41598-022-24316-0)
Supplement: Supplementary file 1 — Supplementary Information. [file 41598_2022_24316_MOESM1_ESM.pdf]

Supplemental Material for *Persistent organic pollutant exposure contributes to Black/White differences in leukocyte telomere length in the National Health and Nutrition Examination Survey*

Emily K. Roberts, Jonathan Boss, Bhramar Mukherjee, Stephen Salerno, Ami Zota, Belinda L. Needham

Supplemental Figure 1. Study Exclusion criteria from NHANES data to final sample size of 1,251 included in the analysis.

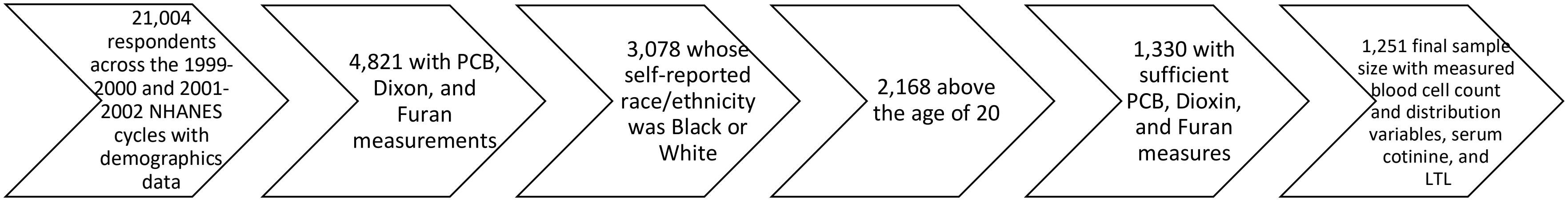

Supplemental Figure 2. Heat map of Spearman pairwise correlation matrix for POPs. These are calculated post-imputation (after calculating a correlation matrix for each imputed dataset and averaging them together). Figure was produced using the *corrplot* package in R (version 0.84).[1]

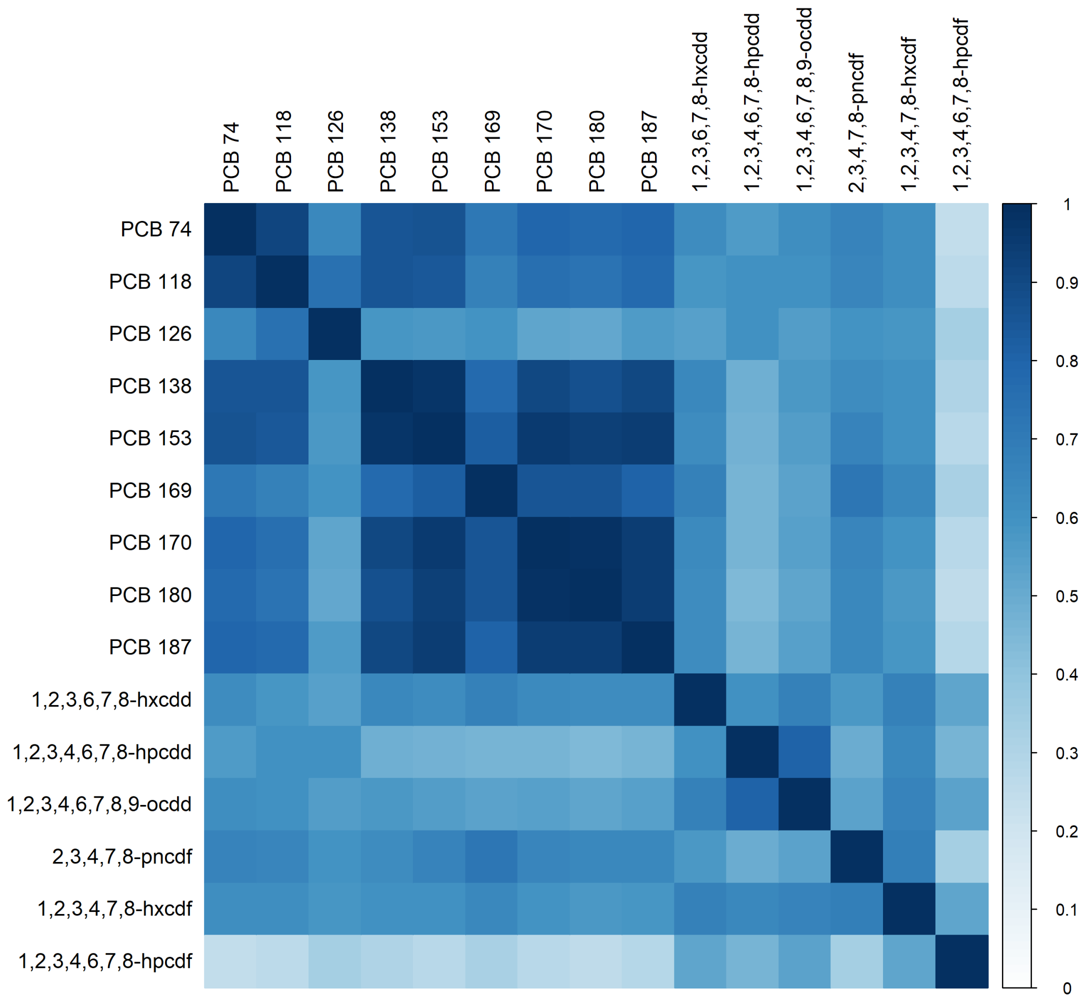

Supplemental Table 1. Point estimates, confidence intervals, and p-values for the total effect of race on LTL, effect of race on mediators, and effect of mediators on LTL from covariate adjusted, single mediator models.

Model	Estimate	CI Lower	CI upper	p-value
**Total effect of race on LTL**	0.054	0.009	0.099	0.018
**Effect of race on mediators**				
Race -> PCB 74	0.117	0.018	0.216	0.020
Race -> PCB 118	0.326	0.210	0.441	<0.001
Race -> PCB 126	0.294	0.142	0.445	0.522
Race -> PCB 138	0.322	0.208	0.436	<0.001
Race -> PCB 153	0.309	0.211	0.406	<0.001
Race -> PCB 169	0.029	-0.059	0.116	<0.001
Race -> PCB 170	0.186	0.099	0.272	<0.001
Race -> PCB 180	0.198	0.111	0.285	<0.001
Race -> PCB 187	0.445	0.347	0.544	<0.001
Race -> D03	0.122	-0.047	0.292	0.157
Race -> D05	0.174	0.072	0.277	<0.001
Race -> D07	0.182	0.079	0.284	<0.001
Race -> F03	-0.020	-0.144	0.104	0.750
Race -> F04	0.124	0.034	0.215	0.007
Race -> F08	0.210	0.097	0.323	<0.001
**Effect of mediators on LTL**				
PCB 74 -> LTL	0.047	0.002	0.092	0.038
PCB 118 -> LTL	0.036	-0.009	0.081	0.111
PCB 126 -> LTL	0.044	0.001	0.087	0.048
PCB 138 -> LTL	0.032	-0.009	0.073	0.128
PCB 153 -> LTL	0.033	-0.010	0.076	0.140

PCB 169 -> LTL	0.052	0.001	0.087	0.016
PCB 170 -> LTL	0.040	-0.003	0.083	0.076
PCB 180 -> LTL	0.042	-0.003	0.087	0.066
PCB 187 -> LTL	0.026	-0.019	0.071	0.259
D03 -> LTL	0.050	0.007	0.093	0.022
D05 -> LTL	0.047	0.002	0.092	0.037
D07 -> LTL	0.047	0.002	0.092	0.038
F03 -> LTL	0.055	0.010	0.100	0.016
F04 -> LTL	0.047	0.002	0.092	0.040
F08 -> LTL	0.047	0.004	0.090	0.033

Supplemental Table 2. Sensitivity analyses of mediation results using a Z-Score-Transformed Average T/S Ratio

Model	IDE	DE	% IDE	95% CI IDE	P-Value IDE
PCB 74	0.023	0.189	10.9%	(-0.003, 0.049)	0.147
PCB 118	0.063	0.149	29.6%	(0.021, 0.105)	0.013
PCB 126	0.033	0.179	15.7%	(0.003, 0.064)	0.070
PCB 138	0.073	0.140	34.2%	(0.030, 0.115)	0.004
PCB 153	0.074	0.138	35.0%	(0.033, 0.115)	0.003
PCB 169	0.006	0.206	2.6%	(-0.018, 0.030)	0.671
PCB 170	0.045	0.167	21.1%	(0.013, 0.077)	0.019
PCB 180	0.039	0.173	18.4%	(0.009, 0.069)	0.028
PCB 187	0.095	0.117	44.8%	(0.047, 0.143)	0.002
D03	0.005	0.207	2.4%	(-0.009, 0.019)	0.551
D05	0.018	0.194	8.6%	(-0.008, 0.044)	0.252
D07	0.016	0.197	7.4%	(-0.008, 0.040)	0.274
F03	-0.005	0.218	-2.6%	(-0.031, 0.020)	0.671
F04	0.021	0.191	10.0%	(-0.007, 0.049)	0.232
F08	0.012	0.200	5.7%	(-0.013, 0.037)	0.435
Unpenalized Linear Regression	0.043	0.148	22.6%	(-0.015, 0.098)	0.116
Ridge Regression	0.053	0.137	27.7%	(0.004, 0.091)	0.032
PCA: All Toxicants	0.077	0.135	36.1%	(0.037, 0.117)	<0.001
TEQ	0.063	0.149	29.9%	(0.021, 0.105)	0.003
PDM	0.001	0.211	0.6%	(-0.005, 0.007)	0.700

References

1. Taiyun Wei and Viliam Simko (2021). R package 'corrplot': Visualization of a Correlation Matrix (Version 0.84). Available from https://github.com/taiyun/corrplot.
